# Depressive Symptoms Longitudinally Mediate the Negative Effects of HbA1c on Memory Change Over 6 Years

**DOI:** 10.1093/geroni/igaa057.540

**Published:** 2020-12-16

**Authors:** A Zarina Kraal, Vicki Ellingrod, Laura Zahodne

**Affiliations:** 1 University of Michigan, Ann Arbor, Michigan, United States; 2 University of Michigan, Ann Arbor, United States

## Abstract

Elevated glycated hemoglobin (HbA1c) has been associated with worse episodic memory in Type 2 diabetes. Prospective links between HbA1c and incident depressive symptoms suggest that behavioral mechanisms may underlie this association. Elevations in HbA1c may lead to depressive symptoms via biochemical changes directly caused by Type 2 diabetes or through distress associated with managing the treatment demands of the disease. This study aimed to determine whether depressive symptoms longitudinally mediate associations between HbA1c and episodic memory over 6 years. Participants (N=2,155) comprised adults aged 51+ with self-reported Type 2 diabetes and clinically elevated HbA1c (≥5.7%) at the time the Health and Retirement Study initiated blood collection. A longitudinal mediation model quantified associations between baseline HbA1c and 6-year change in episodic memory through 4-year change in depressive symptoms, controlling for baseline socio-demographics, other health conditions, and medication adherence. HbA1c was assayed from dried blood spots. Depressive symptoms were self-reported twice over four years. Episodic memory, assessed three times over six years, was a z-score composite of immediate and delayed recall of a word list. Increased depressive symptoms four years after baseline partially mediated the negative association between baseline HbA1c and 6-year memory decline. Depressive symptoms partially mediated the concurrent association between HbA1c and memory at baseline. This longitudinal study provides evidence that the deleterious effects of HbA1c on subsequent episodic memory may operate partly through behavioral mechanisms. Depressive symptoms may represent a critical target for interventions to reduce the enduring negative effects of hyperglycemia on memory aging in Type 2 diabetes.

